# Cardiac dyspnea risk zones in the South of France identified by geo-pollution trends study

**DOI:** 10.1038/s41598-022-05827-2

**Published:** 2022-02-03

**Authors:** Fanny Simões, Charles Bouveyron, Damien Piga, Damien Borel, Stéphane Descombes, Véronique Paquis-Flucklinger, Jaques Levraut, Pierre Gibelin, Silvia Bottini

**Affiliations:** 1 Center of Modeling, Simulation and Interactions, Université Côte d’Azur, 151 Route de Saint Antoine de Ginestiere, 06200 Nice, France; 2 Inria, CNRS, Laboratoire JA Dieudonné, Maasai Research Team, Université Côté d’Azur, Nice, France; 3 ATMOSud, Nice, France; 4 Innovation E-Santé Sud, Hyères, France; 5 Inria, CNRS, Laboratoire JA Dieudonné, Université Côté d’Azur, Nice, France; 6 Inserm U1081, CNRS UMR7284, IRCAN, CHU de Nice, Université Côte d’Azur, Nice, France; 7 Département Hospitalo-Universitaire de Médecine d’Urgence, Nice, France; 8 Faculté de Médicine, Université Côte d’Azur, Nice, France

**Keywords:** Cardiology, Risk factors, Health care, Disease prevention, Health policy, Public health, Quality of life, Weight management

## Abstract

The incidence of cardiac dyspnea (CD) and the distribution of pollution in the south of France suggests that environmental pollution may have a role in disease triggering. CD is a hallmark symptom of heart failure leading to reduced ability to function and engage in activities of daily living. To show the impact of short-term pollution exposure on the increment of CD emergency room visits, we collected pollutants and climate measurements on a daily basis and 43,400 events of CD in the Région Sud from 2013 to 2018. We used a distributed lag non-linear model (DLNM) to assess the association between air pollution and CD events. We divided the region in 357 zones to reconciliate environmental and emergency room visits data. We applied the DLNM on the entire region, on zones grouped by pollution trends and on singular zones. Each pollutant has a significant effect on triggering CD. Depending on the pollutant, we identified four shapes of exposure curves to describe the impact of pollution on CD events: early and late effect for NO_2_; U-shape and rainbow-shape (or inverted U) for O_3_; all the four shapes for PM10. In the biggest cities, O_3_ has the most significant association along with the PM10. In the west side, a delayed effect triggered by PM10 was found. Zones along the main highway are mostly affected by NO_2_ pollution with an increase of the association for a period up to 9 days after the pollution peak. Our results can be used by local authorities to set up specific prevention policies, public alerts that adapt to the different zones and support public health prediction-making. We developed a user-friendly web application called Health, Environment in PACA Region Tool (HEART) to collect our results. HEART will allow citizens, researchers and local authorities to monitor the impact of pollution trends on local public health.

## Introduction

Cardiac dyspnea (CD) is a hallmark symptom of heart failure leading to reduced ability to function and engage in activities of daily living^1^. This pathology particularly affects people aged more that 65 years old with a slight higher prevalence rate in women^2^. Effective management of CD awaits a better understanding of its triggering features. Although cardiovascular factors are believed to play an important role, the experience of CD is multifactorial and may originate from different sources including environmental factors^3^.

A growing body of evidence states that air pollution is a significant threat to health worldwide. The duration of the period of exposure to air pollution yields multiple consequences on health. A short-term exposure increases hospital admissions and mortality rate, causing mainly respiratory and cardiovascular diseases, including CD^4–6^, whereas a long-term exposure reduces life expectancy^7–11^.

Bourdrel et al.^12^ showed that long-term exposure leads to an average increase of 11% in cardiovascular mortality for an annual increase of 10 µg/m^3^ in particulate matter less than 2.5 diameter (PM2.5). Increased hospitalizations and risk of mortality upon chronic exposure to PM2.5 was also found in two epidemiological studies^13,14^. Similarly, cardiovascular mortality increases for long-term and short-term exposures at dioxide nitrogen (NO_2_)^15^. Ozone (O_3_) also has effects on health^16^. Raza et al.^17^ highlighted that short-term exposure to O_3_ is associated with a high risk of cardiac arrest.

Although the relationship between short-term exposure to air pollution and several cardiovascular pathologies, such as acute myocardial infarction and congestive heart failure^18^, is widely established; the link regarding CD is not yet fully demonstrated.

The aim of this study is to shed a light on CD causation mechanisms and to develop prevention policies for health care by combining pollutants, environmental factors and CD hospitalization events.

## Methods

### Study design

**Figure 1:**
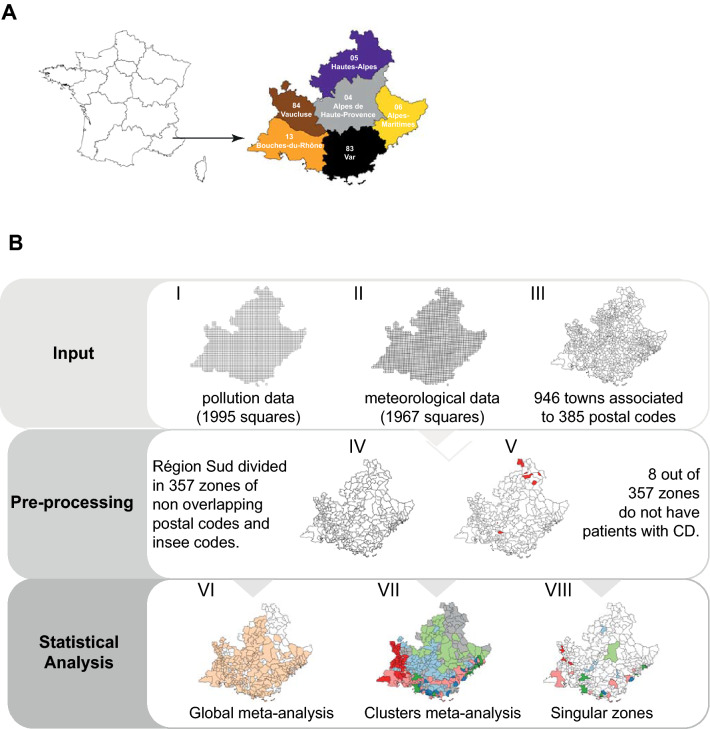
Study design and data analysis workflow. **(A)** The Région Sud and its 6 departments. The three databases used had different resolutions: non-overlapping meshes of 4 km squares for pollution **(B I)** and meteorological variables **(B II)**, and postal codes for emergency room visits data **(B III)**. To reconciliate the databases, we created 357 zones **(B IV)** of non-overlapping postal codes. 8 zones, for which no CD events were registered during the period under study, were filtered out, for a total of 349 zones **(B V)**. Finally, the model was run globally on 251 zones out of 349 because 97 zones contain less than 18 CD events (minimal empirical value of employability of the model) over the entire period of 6 years concerned by this study **(B VI)**. Then the model was run on zones grouped by pollution trends **(B VII)**. Finally, the model was run on the 251 zones singularly and 23 zones with the highest number of CD events were selected **(B VIII)**. Maps were created with the R package maptools v1.1-1.

The Région Sud (previously known as Provence-Alpes-Côte d'Azur, PACA region) is one of the eighteen administrative regions of France with a population of around 5 millions of people covering a territory of 31,400 km^2^. It encloses six departments: Alpes-de-Haute-Provence (04), Alpes-Maritimes (06), Bouches-du-Rhône (13), Hautes-Alpes (05), Var (83) and Vaucluse (84) (Fig. 1A). The peculiarity of this region is that it presents a wide variety of landscapes: the Alps mountains, plains and coastal zones where the majority of the population lives. The region is subjected to different pollutants with extreme local specificity due to different distribution of industrial and agricultural activities, urbanization and traffic all over the territory. Furthermore, a demographic imbalance is also present, with 1 out of 4 citizens older that 60 years old. Altogether these characteristics make the Région Sud a suitable model to study the relationship between CD and pollution.

### Emergency room visits and environmental data

Our study is based on two different databases. The emergency room visits data, provided by the agency ORUPACA (https://ies-sud.fr/) that consists of 43,400 recorded events for individuals aged between 20 and 115 years old, who attended one of the 47 emergency centers of the Région Sud with symptoms related to CD between 2013 and 2018. Inclusion criteria include only patients with International Classification of Disease, version 10, (ICD-10, https://icd.who.int/browse10/2019/en#/) emergency codes: “I50” for heart failure and “I48” for atrial fibrillation and flutter (Table 1). We decided to include atrial fibrillation and flutter and not acute coronary syndrome because an atrial dysrhythmia is a frequent cause of heart failure decompensation. On the other hand, acute coronary syndrome presentation is rarely an isolated dyspnea and the main clinical presentation is chest pain, even if ischemia might be the cause of cardiac decompensation. Pre-anonymized patient data are available, including age, gender and residential postal code (Table 2).

**Table 1:** Inclusion codes for patient population.

Emergency code	Explication	Number of events
I50 = I50.0, I50.1, I50.9, I500, I501, I509	Heart failure	42,769
I48 = I48.0, I48.1, I48.2, I48.3, I48.4, I48.9 associated with codes: J18.9, T17.9, R06.0, T75.1	Atrial fibrillation and flutter	631

**Table 2:** Main characteristics of the study population in Région Sud from 2013 to 2018.

Characteristics	Total	2013	2014	2015	2016	2017	2018
**Gender, n (%)**							
Male	19 991 (46)	2 660 (45)	2862 (46)	3089 (46)	3373 (46)	3926 (46)	4081 (47)
Female	23,409 (54)	3261 (55)	3406 (54)	3575 (54)	3933 (54)	4542 (54)	4692 (53)
Age, mean	82	82	82	82	82	82	82
**Age group, n (%)**							
20–59	1784 (4)	250 (4)	258 (4)	302 (4)	308 (4)	339 (4)	327 (4)
60–69	3468 (8)	459 (8)	502 (8)	519 (8)	585 (8)	695 (8)	708 (8)
70–79	7984 (18)	1123 (19)	1171 (19)	1193 (18)	1325 (18)	1535 (18)	1637 (19)
80–89	18,947 (44)	2661 (45)	2837 (45)	2933 (44)	3269 (45)	3610 (43)	3637 (41)
> 90	11,217 (26)	1428 (24)	1500 (24)	1717 (26)	1819 (25)	2289 (27)	2464 (28)
**Department, n (%)**							
04	2300 (5)	336 (6)	338 (6)	351 (5)	380 (5)	397 (5)	498 (6)
05	1196 (3)	227 (4)	193 (3)	170 (3)	200 (3)	197 (2)	209 (2)
06	9608 (22)	1214 (21)	1339 (21)	1486 (22)	1674 (23)	1824 (21)	2071 (24)
13	12,501 (29)	1628 (27)	1772 (28)	1770 (27)	2108 (29)	2589 (31)	2634 (30)
83	11,968 (28)	1786 (30)	1743 (28)	1985 (30)	1961 (27)	2294 (27)	2199 (25)
84	5827 (13)	730 (12)	883 (14)	902 (13)	983 (13)	1167 (14)	1162 (13)

Environmental data, provided by the agency AtmoSud (https://www.atmosud.org/), are composed by daily pollution and meteorological factors measurements over the period from 2013 to 2018. AtmoSud relies on a set of eighty static and mobile sensors which measure daily pollutants and meteorological variables. Regarding the pollutants, hourly measurements are taken during the day, but only the maximum value for ${NO}_{2}$, and ${O}_{3}$, and the average value for PM10 are registered. Then, using the sophisticated model Chimere^19^ to interpolate sensors measurements, a detailed distribution map with a resolution of 4 km for each of these variables is released. Specifically, the region is divided in squares of 4 km, each square represents a measure, constituting a mesh of 1995 and 1967 squares for pollution and meteorological variables respectively. The environmental data are composed by 3 pollutants: the daily maximum observed of NO_2_ and O_3_ and the daily average value of PM10, and 4 meteorological factors, namely: the daily maximal observed temperature (T) and pressure (P) and the difference between the maximal and minimal value ($\Delta T$ and $\Delta P$).

### Pre-processing and merging

#### Missing values and outliers of environmental data

The environmental database contained missing data, specifically there were 43 days missing (2%) for the pollution data and 22 (1%) for the meteorological data over 2,191 days between 2013 and 2018. Outliers were also detected and were processed as missing data. An outlier is an abnormal value, namely a value that can impossibly be feasible for the associated variable. Two methods were used: interpolation and random sampling. The interpolation method was used for variables with a trend or seasonality (O_3_, PM10, P, T) and the random sampling method for variables with a random distribution (NO_2_), see Supplementary Information for details.

#### Merging emergency room visits and environmental data

The three databases had different resolutions: non-overlapping meshes of 4 km squares for pollution (Fig. 1B, insert I) and meteorological variables (Fig. 1B, insert II), and postal codes for emergency room visits data (Fig. 1B, insert III). In order to associate pollutant and meteorological measurements to each emergency room visit, we first needed to change the data resolution of the three databases. Thus, we created 357 zones (Fig. 1B, insert IV) of non-overlapping postal codes with associated a vector of 10 variables, namely: patient id, age, gender, maximal value for NO_2_, O_3_, T, P, daily temperature amplitude ($\Delta {\varvec{T}}$) and daily pressure amplitude ($\Delta {\varvec{P}}$) and the average value for PM10. Finally, we filtered out 8 zones for which no CD events were registered during the period under study, for a total of 349 zones (Fig. 1B, insert V). Once the bases were reconciled, each emergency room visit has been associated to the environmental data of its residential zone. The database reconciliation procedure is fully explained in Supplementary Information.

### Statistical analysis

We employed a distributed lag non-linear model (DLNM)^20^. The DLNM allows to take into account the non-linear and delayed effects of environmental variables on CD events. It is usually employed for time series data. It is based on the definition of a « cross-basis » function, it describes simultaneously the shape of the relationship along both the space of the predictor and the lag dimension of its occurrence. We defined the cross-basis functions of environmental variables and their lags as natural cubic splines in order to take into account the trend and seasonality of the data. To choose the values of the parameters, we used as reference the zone corresponding to the city of Marseille because it has the highest number of CD events during the studied period. We tested several values of degree of freedom, between 2 and 5, for environmental variables and their lags, and 2 to 8 for time function. Among the meteorological variables, to avoid correlated variables, we included in our model only the maximum of amplitude of pressure and temperature and the maximum of temperature and pressure or the average of pressure and temperature. The selection was done according to the minimization of Q-AIC. The degree of freedom selected are: time function (2), ${NO}_{2}$ (2), PM10 (3), ${O}_{3}$ (3), T (2), P (4), ΔT (2), ΔP (2). The logarithm of the number of people for each zone (called here “population”) is also included in the model. The number of habitants by INSEE code is found on website “*notreterritoire.maregionsud.fr*” and we calculated the population for our defined 357 zones. Since data were available only for the years up to 2016, we assumed the population to be constant for 2017 and 2018.

The association between environmental variables and CD events is studied on a period of 14 days (lags). The model applied is a generalized linear model with quasi-Poisson distribution. The explained variable is the number of CD by date and by zone. We represented the association between the outcome and predictors by the relative risk (RR) or overall effect for a specific increase above the threshold at each lag. Specifically, for each pollutant, we selected four levels of exposure from the World Health Organization (WHO) (https://www.who.int/) air quality guidelines, namely: “good”, “moderate”, “lightly”, “heavily” (intervals are indicated in Table 3). Then we applied the statistical model based on DLNM to assess the RR for each of the four defined level when data are available for the studied period, compared to the basal reference value for each pollutant, for a period of 14 days before the event. For meteorological variables we divided the distribution in four percentiles (1st, 10th, 90th, 99th) and the RR was assessed compared to median value (intervals are indicated in Table 4). Since both high and low temperature can have an effect on the RR, to measure this association we chose the median because by definition is the value separating the higher half from the lower half of data distribution.

**Table 3:** Pollutant threshold ranges associated to selected levels for this study from the WHO guidelines.

Levels of pollution	NO_2_ (μg/m^3^)	O_3_ (μg/m^3^)	PM10 (μg/m^3^)
Reference (very good)	50	40	10
Good	61–120	55–108	16–30
Moderate	121–140	109–126	31–35
Lightly	141–200	127–180	36–50
Heavily	> 200	> 180	> 50

**Table 4:** Meteorological variable threshold ranges associated to selected levels for this study.

Meteorological levels	Temperature (°C)	Pressure (hPa)	Temperature amplitude (°C)	Pressure amplitude (hPa)
1st percentile	0.5–10.4	1001.2–1011.7	2.2–6.1	1.4–4
10th percentile	5–12.9	1010.1–1020.5	4–8.2	2–5.2
50th percentile Median	16.1–20.6	1017.8–1028	6.1–11.9	3.7–7.2
90th percentile	25.5–31.5	1026.8–1037.1	8.8–15.4	8.2–11.6
99th percentile	29.6–35.8	1035.1–1045.5	12–17.7	15.2–18.9

Levels may overlap due to the variability of the distributions on each cluster or zone.

Singular pollutant model includes one pollutant and meteorological variables whereas multivariate pollutants model includes all pollutants and meteorological variables. In total, 8 models are tested (6 singular and 2 multivariate). The degree of freedom of pollutant variables (${NO}_{2}$, ${O}_{3}$, PM10) are fixed according to singular pollutant model whereas the degree of freedom of meteorological variables are fixed according to multivariate pollutants model. Since singular and multivariate pollutants models gave similar results, only the last one is retained for analysis.

### Multivariate meta-analysis

We employed a multivariate meta-analysis in order to synthesize results in multi-zones analysis with DLNM. It defines an average exposure–response association across the zones selected. The method of estimation for random effect multivariate meta-analysis used is restricted maximum likelihood (reml). The R package used is *mixmeta*.

This model was applied at three level on our data. Before running the model, we removed 97 zones having less than 18 CD events (minimal empirical value of employability of the model).

### Global model on the entire region

We performed a multivariate meta-analysis with the DLNM^21^ on the previously defined zones in order to identify global trends on the risks of CD by pollutants at the region level (Fig. 1B, insert VI).

### Global model on the zones grouped by pollution trends

To take into account local fine structures of pollution distribution, we employed the co-clustering algorithm *“multiFunLBM”*^22^ on the environmental dataset in order to identify groups of zones with homogeneous trends of pollutions (Fig. 1B, insert VII). To define groups of zones with similar trends of pollution, we used a specific co-clustering method developed for time series with R package *funLBM*. The algorithm takes into account 2 dimensions: space and time. The analysis is done by week of 7 days (313 weeks for the 6 years). The aim is to identify zones and weeks having similar trends of environmental variables distributions. Parameters used are: the basis function is Fourrier, commonly used function for time series of periodic data, the type of initialization is funFEM. According to the Integrated Completed Likelihook (ICL) criterion, 6 clusters are obtained. The cluster 1 corresponds to countryside (69 zones), cluster 2 to coast-side/medium size cities (23 zones), cluster 3 to mountains (31 zones), cluster 4 to coast-side/ big cities (17 zones), cluster 5 to highway (58 zones) and cluster 6 to west side (53 zones). Finally, we applied the same model at the pollution clusters level.

### Local model on the singular zones

In order to identify specific local trends, we applied the DLNM singularly on each zone (Fig. 1B, insert VIII). Finally, we selected 23 zones with the highest number of CD events, representatives of each pollution cluster to explore the relationship between pollution and CD at the local level.

### Web application HEART

In order to collect and to easily access to the results of this study, we developed a web application called HEART: Health-Environment PACA Region Tool. HEART can be easily downloaded at https://github.com/UCA-MSI/HEART.

## Results

### Overall characteristics of emergency room visits data and environmental database

The emergency room visits data is composed by 43,400 CD events collected by the 47 emergency centers of Région Sud in the period from 1st January 2013 to 31st December 2018 reporting an event of CD with symptoms related to either heart failure (I50 codes, 98% of the events) or atrial fibrillation (I48 codes, 2% of the events) (Table 1). A summary of emergency room visits data characteristic is reported in Table 2.

**Figure 2:**
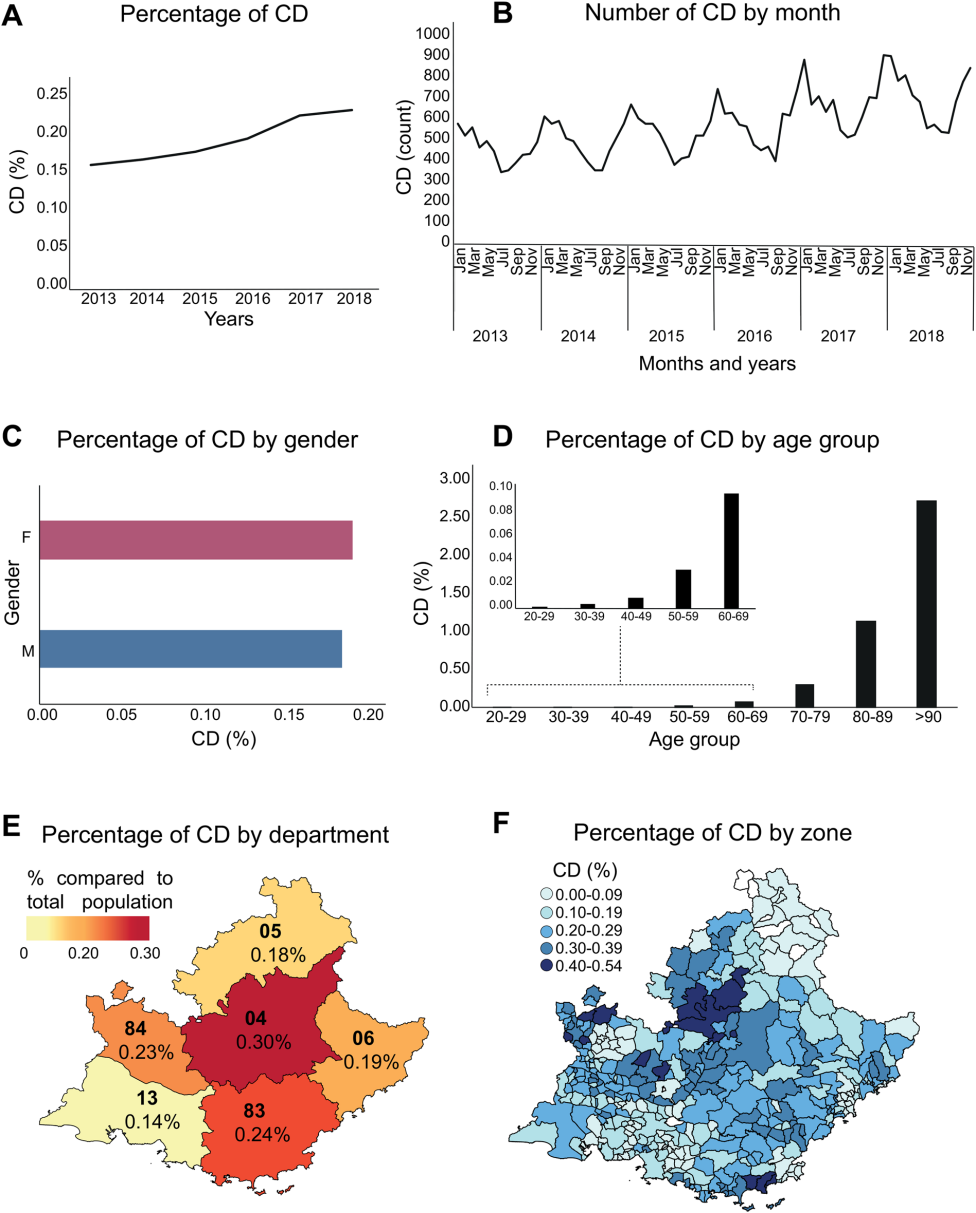
Main characteristics of emergency room visits data. The incidence of CD events in Région Sud **(A)**, by season **(B)** and by gender **(C)**. The distribution of CD events by age **(D)** by department **(E)** and by zone **(F)**. Maps were created with the R package maptools v1.1-1.

We normalized the number of events by the total population by year and by zone and we reported the incidence of CD events in Région Sud in Fig. 2A. Indeed, only a small percentage of the population is concerned, slightly increasing over the period of the study. As expected, we observed a higher incidence of CD events in wintertime compared with summer (Fig. 2B) and no specific prevalence due to gender (Fig. 2C). The distribution of CD events by age follows the expected pattern: very few events among people younger than 70 years old with a narrow peak for people aged more than 90 years old (Fig. 2D). Finally, the distribution of CD events by department and by zone are reported in Fig. 2E,F, respectively. Overall similar percentages are observed, specifically the department of Alpes-de-Haute-Provence (04) located to the Alps reported the highest percentage of events (0.30%), along with the department of Var (83) on the coast (0.24%) while the lowest percentage is observed in the department of Bouches-du-Rhône (13) (0.14%).

The characteristics of environmental data are summarized in supplementary table 1 and time series trends for each pollutant and meteorological variables in Région Sud in 2013 to 2018 are showed in Supplementary Fig. 1. We observe that O_3_ has a periodic behavior with peaks during summertime, PM10 has a less strong seasonality and NO_2_ does not show any periodicity. As expected, temperature has a seasonal pattern, while pressure shows a month periodic trend. No particular trend is reported for temperature and pressure amplitudes. The average values of each pollutant by zone, reported in Supplementary Fig. 2, highlight the strong spatial distribution of each pollutant in the region. The correlations between air pollution and meteorological factors are presented in Supplementary Fig. 3. The daily concentrations of O_3_, NO_2_, and PM10 are not correlated. Similarly, poor correlation between pollutant levels and meteorological factors is reported with exception of a good and positive correlation between O_3_ and T (correlation coefficient r = 0.69, P < 0.05), as expected. Finally, none of the environmental variable correlates with CD. The absence of correlation among the variables allow the use of the distributed lag non-linear model (DLNM) to identify the association between pollution and CD events.

### Global model on the entire region finds significant associations between meteorological factors and CD events

We applied the DLNM model globally on the entire region and we measured the relative risk (RR) of having a CD event after exposure to pollution and meteorological factors (see “Methods” for details on the model set-up). Results are reported by exposure curves to show global trends and by single day lag-response confidence intervals (CI) to show significance of RR.

Overall, no significant associations were found for NO_2_ and PM10 (Supplementary Fig. 4A,C, respectively) on RR of CD when the model is applied globally on the entire region. Slightly significant associations are observed for O_3_ when levels raise above “heavily polluted” compared to reference level, increasing the RR up to 1.06 (95% CI: 1–1.15), appearing at lag 0 and decreasing until no significant at lag 3 (Supplementary Fig. 4B). These results suggest that the global model is not suitable for our data due to the different local distribution of pollution all over the region.

**Figure 3:**
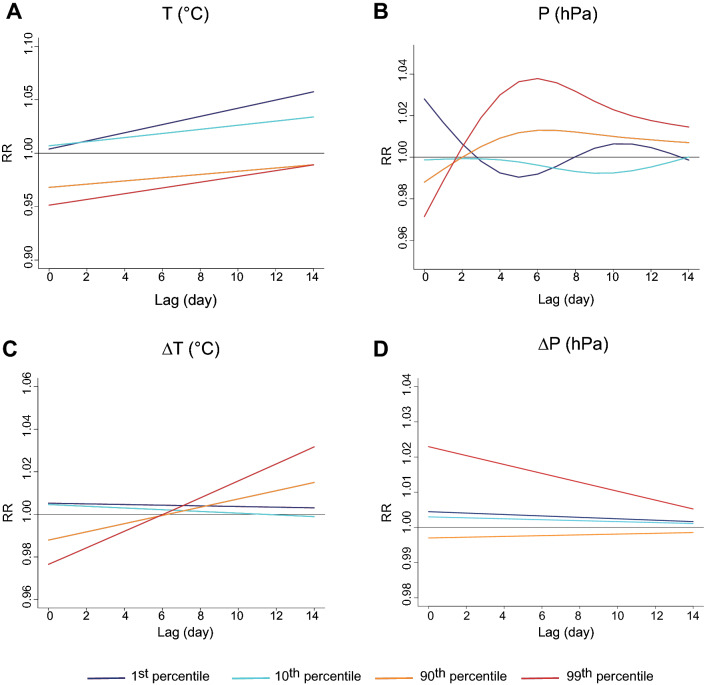
Global DLNM (only significant results are reported). Lag-specific effect on CD events for increases of each of the meteorological variables temperature **(A)**, pressure **(B)**, temperature amplitude **(C)** and pressure amplitude **(D)**, over the thresholds indicated in the legend in the plot for all zones in Région Sud.

On the other side, regarding meteorological variables, the global model showed good performances, due to a more homogeneous distribution over the territory. As expected, low temperatures (1st percentile) increase the RR of CD progressively from 1.01 (95% CI: 1.00–1.05) at lag 3 up to 1.04 (95% CI: 1.02–1.06) at lag 14 (Fig. 3A and Supplementary Fig. 4D) as well as high pressures (99th percentile) from lag 3 up to lag 12 [RR between 1.02 (95% CI: 1.00–1.04) and 1.04 (95% CI: 1.01–1.06)] (Fig. 3B and Supplementary Fig. 4E). High values of temperature amplitude (above the 99th percentile) increase RR of CD up to 1.04 (95% CI: 1.00–1.06) from lag 10 to lag 14 (Fig. 3C and Supplementary Fig. 4F). Finally, a slight association is observed only for high values of pressure amplitude (99th percentile) at lag 0 yielding a RR of 1.02 (95% CI: 1.00–1.04) up to lag 10 (Fig. 3D and Supplementary Fig. 4G). Overall, low temperature, high pressure and high amplitude values for these two parameters have a significant association on the RR of CD.

### Global model by pollution clusters identified significant associations between pollution and CD events

**Figure 4:**
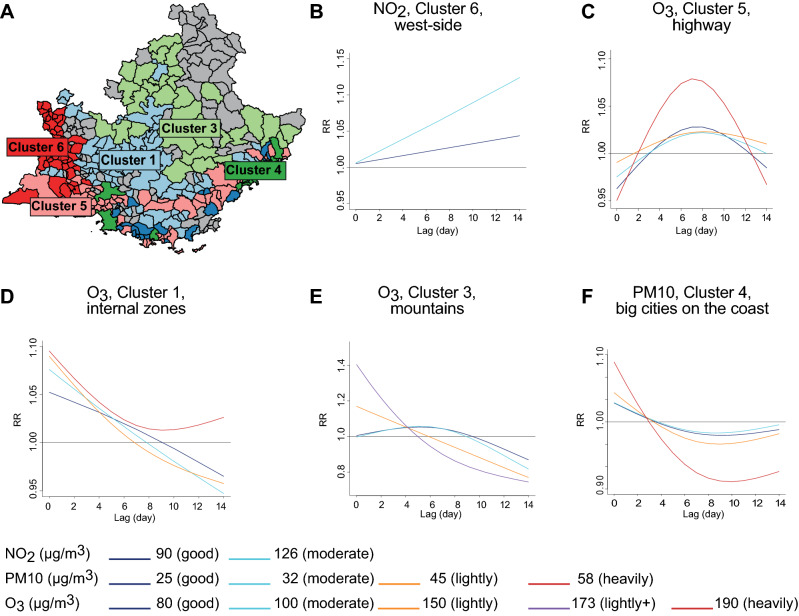
DLNM by pollution clusters (only significant results are reported). **(A)** Zones of Région Sud coloured by cluster of pollution. Colour code: light blue, cluster 1 (internal zones on the mountains); light green, cluster 3 (mountains); dark green, cluster 4 (big cities on the coast); pink, cluster 5 (highway); red, cluster 6 (west side); grey, zones not included. **(B)** Lag-specific effect on CD events for increases of NO2 levels over the two thresholds as indicated in the legend in the plot for zones in cluster 6 (west side). Lag-specific effect on CD events for increases of O_3_ levels over the thresholds indicated in the legend in the plot for zones in cluster 5 (highway) **(C)**, cluster 1 (internal zones on the mountains) **(D)** and cluster 3 (mountains) **(E)**. **(F)** Lag-specific effect on CD events for increases of PM10 over the thresholds indicated in the legend in the plot for zones in cluster 4 (big cities on the coast). Maps were created with the R package maptools v1.1-1.

As already showed, the global model did not find any conclusive relationship between pollution exposure and CD events. We reasoned that the different territorial distribution of pollutants was not properly considered in the global analysis (Supplementary Fig. 2). Thus, we took advantage of our previous work on the analysis of the environmental data in Région Sud^22^, in which we grouped the 357 zones in 6 clusters having homogeneous trends of pollution (Fig. 1B, insert VII and Fig. 4A), to exploit the impact of pollution on CD events. Curves of exposure and CI for significant RR values by lag for each cluster and pollutant are reported in Fig. 4 and supplementary table 2, respectively.

Exposure curves for NO_2_ show a significant impact on RR only for cluster 6, that collects zones of the west side of the region, when the level overcomes the heavily polluted threshold, with linear trend increasing by lag (Fig. 4B).

For cluster 5, the association on RR becomes slightly significant with the increasing of pollution levels between lag 6 and lag 10 (Fig. 4C). An immediate association up to lag 2 is observed when O_3_ level raises above lightly for cluster 1 (internal zones on the mountains) (Fig. 4D) and lightly + for cluster 3 and up to lag 4 when O_3_ level raises above good, moderate (Fig. 4E).

PM10 has the highest impact on RR in cluster 4 with an immediate association at lag 0 increasing significantly the RR up to 1.1 (95% CI: 1.00–1.3) regardless the level of pollution (Fig. 4F).

Overall, these results pointed out that RR of CD is affected by pollution levels, with trends and lags that depends by local territorial characteristics.

### Local model by singular zones identified specific exposure curves trends

**Figure 5:**
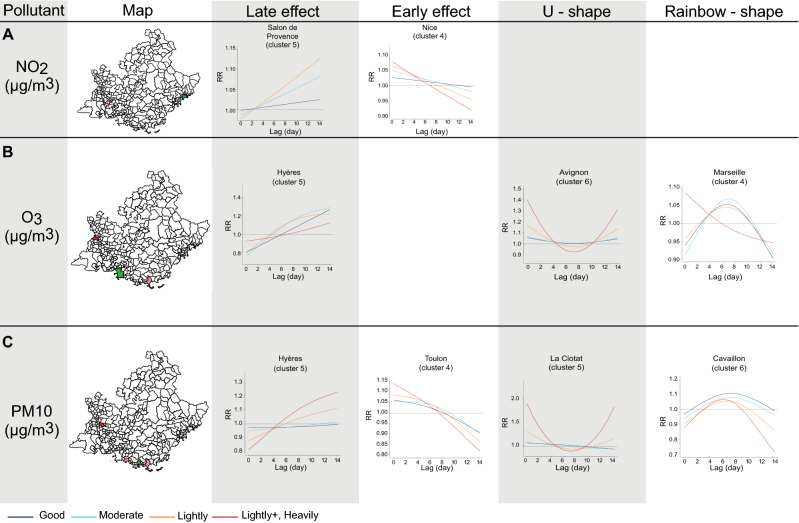
DLNM by singular zones, only significant results for the selected 23 zones are reported. Lag-specific effect on CD events for increases of NO_2_ **(A)**, O_3_ **(B)** and PM10 **(C)** over the thresholds indicated in the legend in the plot. Only the most representative plot is reported for each pollutant and shape of the response-effect curves. Others zones showing similar trends are reported in Supplementary Fig. 5. Maps were created with the R package maptools v1.1-1.

Previous results, although significant, are mild. In order to exploit more how local pollution affects the RR of CD, we applied the DLNM statistical model singularly to each of the 251 zones of Région Sud with at least 18 CD events. Then we selected 23 zones with the highest number of CD events in order to guarantee a geographic representativity of the region peculiarities. Results for pollution variables on these zones are reported in Fig. 5, supplementary Fig. 5 and supplementary table 3. The study of several zones allowed the identification of common patterns of exposure curves characteristics of each pollutant. Therefore, four cumulative exposure curves shapes were identified: early and late effect for NO_2_; rainbow-shape (inverted U) and U-shape for O_3_; the four for PM10 (Fig. 5, supplementary Fig. 5).

NO_2_ has a significant association with CD emergency room visits only in some zones belonging mainly to cluster 5, namely: “Grasse”, “Le Luc” (supplementary Fig. 5A), and mildly for “Salon de Provence” with two exposure patterns, namely “early effect” and “late effect” (Fig. 5A). This result is consistent with the characteristics of NO_2_ pollution, mainly due to fuel combustion from motor vehicles and present in densely populated zones, and of the zones collected in these clusters passing by the main highway of the region. Notably, only for the zone “Nice” of cluster 4, NO_2_ increases over the level heavily polluted yielding a RR of 1.08 (95% CI: 1.00–1.15) at lag 0 (Fig. 5A). The presence of the airport along with densely populated area with elevated traffic might support this result.

Two main exposure patterns were observed for O_3_ (Fig. 5B, supplementary Fig. 5B): rainbow-shape (“Draguignan”, cluster 1; “Marseille”, “Aix en Provence” and “Toulon”, cluster 4; “Grasse”, cluster 5) or U-shape (“St. raphael”, cluster 2; “Salon de Provence”, cluster 5; “Avignon”, cluster 6). We observed that, often the rainbow-shape curves switch to U-shape trends when O_3_ level increases over heavily polluted thresholds (“Draguignan”, cluster 1 and “Toulon” supplementary Fig. 5B, cluster 4; “Marseille” , cluster 4, Fig. 5B). This switch could be due to an exacerbation of the association on the pathology going from delayed effect to immediate effect because the pollutant is not tolerated anymore by the individuals. This result suggests that the higher the O_3_ pollution levels are, the earlier the association with CD events is significant. The zone “Hyères” in cluster 5 shows a late effect profile, with a significant association from lag 7 up to lag 14 when O_3_ levels are higher than good, moderate and lightly polluted (Fig. 5B).

Several different exposure profiles were identified for PM10 (Fig. 5C, supplementary Fig. 5C): early effect (“Marseille”, “Nice” and “Toulon”, cluster 4; “Salon de Provence”, cluster 5; “Avignon”, cluster 6), rainbow-shape (“Gap”, cluster 1; “Cavaillon”, cluster 6), U-shape (“La Farlède”, cluster 2; “La Ciotat”, cluster 5) and late effect (“Pertuis”, cluster 1; “Hyères”, cluster 5).

## Discussion and conclusions

This is the first study of pollution associations on CD events done in Région Sud and, as far as we know, the first study to cover an entire region. We collected pollutants (NO_2_, O_3_, and PM10) and climate measurements (Temperature and Pressure) on a daily basis and 43,400 events of CD in the Région Sud from 2013 to 2018. We present evidences that the three pollutants are associated with significant increment in CD emergency room visits. Four shapes of exposure curves were identified: early and late effect for NO_2_; rainbow-shape (inverted U) and U-shape for O_3_; all the four shapes for PM10. Importantly, our approach effectively proposes pollutants monitoring as tool for prediction-making for public health policies.

In this study, we have found significant associations between short-term exposure to NO_2_, O_3_, PM10 and CD events at the zone level. The strongest effect on CD incidence was observed for PM10, as for previous studies reported on other cardiovascular illnesses^23–25^. This finding is supported by biological studies suggesting that particulate matter tends to deposit in pulmonary alveoli more than gaseous pollutants, triggering extra-pulmonary effects^13^. Our study reported a positive association of CD events with O_3_, as already showed for acute coronary events^26^. We observed a weak relationship between NO_2_ and CD, as previously showed for acute myocardial infarction failure^18–24^.

We found that not only extreme pollution levels could increase the risk of CD, but also moderate and good levels depending on the zone of the region. Importantly, the DLNM model used, depicted the non-linear and delayed influence of pollution exposure on CD events. Indeed, we observed four different shapes of exposure curves describing the relationship between pollutants and CD incidence, with important consequences for prevention and treatment. The early-effect curves suggest that the RR increases immediately after exposure and decreases after a certain period until no association on CD is observed. Contrarily, for late effect curves, the opposite scenario is to be expected. U-shaped relationship means that both immediate and late effects on the RR of CD events should be expected. Rainbow-shaped curves yield a slightly delayed effect on CD after the pollution peak, that persists for a certain period (depending on the amplitude of the significant lags). Accordingly, specific alerts can be set up by local authorities to prevent CD events.

One of the limitations of this study could be the resolution of the results: according to the General Data Protection Regulation, only patients’ residential postal code was available although data are anonymized. Thus, in order to associate pollution measurements to each emergency room visit, we had to change the resolution of pollution data from a mesh of 4 km squares to the zone level, yielding merged pollution values for zones covered by more than one square. This may have led an underestimation of exposure associations. However, since most of the zones matched with only one square, data aggregation is expected to play only a minor role in this underestimation. For the same reason, we cannot distinguish repeated emergency room visits from singular events. Multiple visits of the same individual would suggests an exacerbation of the pathology and might be affected by pollution differently. However the period covered by this study is relatively short to have several repeated visits for CD except for chronic CD that are excluded since they have a different ICD-10 code.

By contrast, some strengths should be discussed. First, the data on CD were collected from all the 47 emergency centers of the region allowing complete coverage and the sample size is quite big to achieve good statistical performances. Secondly, the availability of the exact date of CD events and daily measurements of pollutants allowed the temporal alignment between air pollution exposure and CD incidence. Thirdly, we set up a statistical model that can be applied at different scales, from the entire region up to single cities/zones.

In order to collect and to easily access to the results of this study, we developed a web application called HEART: Health-Environment PACA Region Tool. HEART can be used by local authorities in order to set up specific policies to lower down pollution or for public alerts when pollution raise above secure levels for the citizens that adapt to the different zones. Furthermore, it can help to identify periods of the years that are particularly affected by pollution, in order to set up alerts and prevention behaviors for each specific zone.

In conclusion, the value of this study goes beyond the results obtained for the Région Sud, because the statistical model we set up, could be expanded to study other regions or zones and/or the impact of pollution on other pathologies.

## Supplementary Information

Supplementary Information.

## Data Availability

Project name: HEART ; Project home page: https://github.com/UCA-MSI/HEART; Operating system(s): Platform independent ; Programming language: R ; Other requirements: R 3.6 or higher; License: e.g. GNU GPL 3.0 ; Any restrictions to use by non-academics: none. The authors declare that the data supporting the findings of this study are available within the article and its supplementary information files.

## Abbreviations

CD: Cardiac dyspnea
RR: Relative risk
DLNM: Distributed lag non-linear model
NO_2_: Dioxide nitrogen
O_3_: Ozone
PM: Particulate matter
