# Supplementary material for: Cardiac dyspnea risk zones in the South of France identified by geo-pollution trends study
Source: Sci Rep. 2022 Feb 3;12:1900. doi: 10.1038/s41598-022-05827-2 (PMC8813995; doi:10.1038/s41598-022-05827-2)
Supplement: Supplementary file 1 — Supplementary Information. [file 41598_2022_5827_MOESM1_ESM.docx]

***Supplementary information about preprocessing and statistical methods***

***Environmental data***

Environmental data is composed of daily pollution and weather factors measurements over the period 2013 to 2018. Pollution data includes 3 pollutants: the daily maximum value of ${NO}_{2}$, and $O_{3}$, and the daily average value of PM10. Meteorological data is composed of 4 variables: maximum of pressure and temperature, minimum of pressure and temperature. Finally, we created two additional variables: the amplitude of pressure and temperature. All measurements have a resolution of 4 km^2^ thus constituting a mesh of 1995 and 1967 for pollution and meteorological variables respectively for the entire region. Pollution and meteorological meshes are non-overlapping.

***Emergency room visits data***

Emergency room visits data is composed of 43,400 recorded events for individuals have attended one of the 47 emergency departments of the Région Sud with symptoms related to cardiac dyspnea (CD) between 2013 and 2018. The age, postal code, gender, diagnostic code and date of event are available for each patient. Inclusion criteria are minimal age of 20 years old and residential postal codes belonging to the Région Sud. Data are pseudoanonimyzed, meaning that no names are available nor the exact address of the patients. Thus, we do not know if the same patient had multiple CD events during the 6 years under study. All events are considered as independent.

***Merging health data and environmental data***

Patient data have the resolution of the postal codes whereas environmental data have a resolution of squares of 4 km^2^. Thus, in order to associate each patient with environmental data, we had to establish a method to reconciliate the different resolutions. Hence, we created 357 zones.

In 2019, there are 946 towns in Région Sud, in France each town is associated to a unique INSEE code (946 INSEE codes) and to a postal code (385 postal codes). It exists as many INSEE codes as towns in France, it is also called “code commune”. However, postal codes could belong to multiple towns. Usually, big towns have multiple postal codes and small towns share same postal codes. We obtained the boundaries associated to the 946 towns from “*OpenStreetmap*” corresponding to the INSEE codes. We grouped towns associated to the same postal code or INSEE code in order to form a new “zone”. The link between each postal code and their INSEE codes is found in website “*datanova.laposte.fr*” from 2018. For example, the town Nice is associated to one INSEE code (06088) and four postal codes (06100, 06200, 06206, 06300), thus the corresponding zone in our dataset is defined as the union of the boundaries associated to the INSEE code and the 4 postal codes.

Finally, we associated each patient to environmental data through our 357 zones. Firstly, we associated pollution and meteorological measurements to the 357 zones. We used function *st_join()* from R package *sf* to associate squares to zones. If a square covers only one zone, then the association is done. If a square overlaps two zones, it is associated to the zone with the biggest overlap. If a zone covers multiple squares, then it is associated with all of them. With these criteria, we associated all squares to zones, however some zones do not have any square associated. Thus, we iterated the procedure for zones without squares associated. We allowed a square to be associated with two zones. Then, if there are still zones without squares, they are associated to adjacent squares. Finally, all zones are associated to at least one square of environmental data.

Once the association is done, we needed to calculate the value of environmental variables in the new resolution. When a square covers only one zone, it takes the measurement available for the corresponding square. Otherwise, when multiple squares are associated with one zone, we needed to recalculate the value of the environmental variables. For maximum of ${NO}_{2}$, $O_{3}$, temperature, pressure and their amplitudes, the values associated to the zone correspond to the maximum value among the measurements for all the squares associated for one variable. For the average of PM10, the value associated to zone is the average value among the squares covered by this zone. Accordingly, for the minimum of temperature and pressure, the values associated to zones are the minimum values among the corresponding squares.

Finally, we associated each patient to the defined zones. We filtered out 8 zones for which no CD events were registered during the period under investigation.

To summarize, our final database is constituted by 349 zones with associated 9 variables, namely: maximum of maximum of ${NO}_{2}$ and $O_{3}$, average of average of PM10, maximum of maximum of pressure, temperature and their amplitudes, minimum of minimum of pressure and temperature. To simplify the notation, the environmental variables are written without writing maximum, minimum or average.

***Missing values treatment***

Random sampling is used for variables with random distribution such as ${NO}_{2}$. To set the size of the window where to sample values to replace the missing data, we determined a window *w* equal to 4 days + *m,* where *m* is 1, 2, 3, 4 or 10 days (that are the sizes of the missing periods in our datasets). Then, we divided by 2 the size of the window *w* to have an equal window where to sample data on the left and right of the missing period to reconstruct. If the window cross other missing values, its size is expanded in order to have *w*/2 days where to sample values on both sides. We used function *sample_n*() of *dplyr* R package, to pick out values in the defined intervals to replace missing values. For example, if only one day is missing then *m*=1 and *w* is equal to 4+1=5. The size of the even window surrounding the missing period is calculated by dividing by 2 the window *w* and round to less, thus in our example *w* = 2. Thus, if one day is missing, its value is replaced by a random pick out of one value 2 days before or after the missing period.

An interpolation method is used for variables with a trend or seasonality (PM10, $O_{3}$, pressure and temperature). We used R function *na.approx()* from R package *zoo*.

Only for pressure, measurements were missing for the 1^st^ January 2013. Since this date correspond to the first measurements of our database, we could not use the previous described approach, thus we assumed that they are equal to those of 2^nd^ January 2013.

### Supplementary Table 1. Main characteristics of the environmental data for the 357 zones in Région Sud from 2013 to 2018.

| Environmental variables | Total | 2013 | 2014 | 2015 | 2016 | 2017 | 2018 |
| --- | --- | --- | --- | --- | --- | --- | --- |
| Pollutants  mean years [min,max] |  | | | | | | |
| NO_2_ (μg/m^3^) | 40  [1,296] | 39  [1,296] | 39  [1,261] | 41  [1,228] | 40  [1,227] | 41  [1,267] | 39  [1,262] |
| O_3_ (μg/m^3^) | 93  [5,299] | 91  [16,224] | 91  [5,299] | 94  [7,299] | 92  [8,223] | 95  [18,192] | 95  [13,224] |
| PM10 (μg/m^3^) | 20  [0,100] | 24  [0,93] | 20  [0,93] | 20  [2,83] | 19  [3,70] | 20  [0,74] | 18  [1,100] |
| Meteorological factors  mean years [min,max] |  | | | | | | |
| T max (°C) | 18  [-15,39] | 17  [-14,36] | 18  [-11,35] | 19  [-11,38] | 18  [-12,35] | 18  [-14,39] | 18  [-15,38] |
| P max (hPa) | 1022  [986,1068] | 1021  [993,1059] | 1020  [997,1052] | 1024  [986,1068] | 1022  [996,1068] | 1023  [1001,1056] | 1021  [996,1057] |
| $\Delta T$ (°C) | 9  [0.2,28] | 9  [0.5,20] | 9  [0.8,20] | 9  [0.2,26] | 9  [0.5,28] | 9  [0.4,25] | 8  [0.4,21] |
| $\Delta P$ (hPa) | 6  [0.7,46] | 6  [0.9,46] | 5  [0.8,45] | 5  [0.8,37] | 6  [0.7,36] | 5  [0.7,45] | 5  [0.7,42] |

### Supplementary Table 2. Effect of different levels of pollution on cardiac dyspnea for significant lags for zones grouped by pollution clusters. Missing values are indicated with “x”. If some levels are missing, when possible, we defined novel intervals: good- and lightly+, corresponding to the maximal value reached by the corresponding pollutant in the specific cluster as indicated in the table.

|  |  |  | NO_2_ | | | O_3_ | | | PM10 | | |
| --- | --- | --- | --- | --- | --- | --- | --- | --- | --- | --- | --- |
| Cluster | N of zones | Pollution levels | Value  (μg/m^3^) | Lag | RR  (95% CI) | Value  (μg/m^3^) | Lag | RR  (95% CI) | Value  (μg/m^3^) | Lag | RR  (95% CI) |
| 1 | 97 | Good | 85 | 0 to 14 | 0.85-1.15 | 80 | 0 to 14 | 0.90-1.20 | 25 | 0 to 14 | 0.90-1.10 |
|  |  | Moderate | x | x | x | 110 | 0 to 14 | 0.90-1.20 | 32 | 0 to 14 | 0.90-1.10 |
|  |  | Lightly | x | x | x | 150 | 0 to 14 | 0.80-1.20 | 45 | 0 to 14 | 0.90-1.10 |
|  |  | Heavily | x | x | x | 190 | 0 to 14 | 0.80-1.30 | 60 | 0 to 14 | 0.80-1.30 |
| 2 | 38 | Good | 90 | 0 to 14 | 0.98-1.10 | 80 | 0 to 14 | 0.80-1.30 | 25 | 0 to 14 | 0.90-1.10 |
|  |  | Moderate | 128 | 0 to 14 | 0.80-1.30 | 110 | 0 to 14 | 0.80-1.30 | 32 | 0 to 14 | 0.90-1.10 |
|  |  | Lightly | x | x | x | 150 | 0 to 14 | 0.80-1.30 | 45 | 0 to 14 | 0.90-1.20 |
|  |  | Heavily | x | x | x | 190 | 0 to 14 | 0.50-2.00 | 48  lightly+ | 0 to 14 | 0.60-1.60 |
| 3 | 70 | Good | 62 | 0 to 14 | 0.75-1.30 | 80 | 0 to 14 | 0.80-1.30 | 25 | 0 to 14 | 0.90-1.20 |
|  |  | Moderate | x | x | x | 110 | 0 to 14 | 0.80-1.30 | 32 | 0 to 14 | 0.90-1.30 |
|  |  | Lightly | x | x | x | 150 | 0 to 14 | 0.50-1.50 | 45 | 0 to 14 | 0.80-1.30 |
|  |  | Heavily | x | x | x | 173  lightly+ | 0 to 14 | 0.50-2.00 | 60 | 0 to 14 | 0.60-1.30 |
| 4 | 22 | Good | 90 | 0 to 14 | 0.97-1.02 | 80 | 0 to 14 | 0.90-1.05 | 25 | 0 to 14 | 0.95-1.10 |
|  |  | Moderate | 130 | 0 to 14 | 0.96-1.02 | 110 | 0 to 14 | 0.90-1.05 | 32 | 0 to 14 | 0.95-1.10 |
|  |  | Lightly | 170 | 0 to 14 | 0.95-1.05 | 150 | 0 to 14 | 0.90-1.10 | 45 | 0 to 14 | 0.90-1.20 |
|  |  | Heavily | 210 | 0 to 14 | 0.90-1.10 | 190 | 0 to 14 | 0.85-1.20 | 58 | 0 to 14 | 0.80-1.30 |
| 5 | 69 | Good | 90 | 0 to 14 | 0.95-1.05 | 80 | 0 to 14 | 0.90-1.10 | 25 | 0 to 14 | 0.90-1.10 |
|  |  | Moderate | 130 | 0 to 14 | 0.90-1.10 | 110 | 0 to 14 | 0.90-1.10 | 32 | 0 to 14 | 0.90-1.10 |
|  |  | Lightly | 157 | 0 to 14 | 0.85-1.15 | 150 | 0 to 14 | 0.85-1.15 | 45 | 0 to 14 | 0.90-1.10 |
|  |  | Heavily | x | x | x | 190 | 0 to 14 | 0.80-1.20 | 60 | 0 to 14 | 0.70-1.10 |
| 6 | 61 | Good | 90 | 0 to 14 | 0.98-1.10 | 80 | 0 to 14 | 0.92-1.10 | 25 | 0 to 14 | 0.92-1.10 |
|  |  | Moderate | 126 | 0 to 14 | 0.90-1.30 | 110 | 0 to 14 | 0.90-1.15 | 32 | 0 to 14 | 0.90-1.10 |
|  |  | Lightly | x | x | x | 150 | 0 to 14 | 0.90-1.15 | 45 | 0 to 14 | 0.90-1.15 |
|  |  | Heavily | x | x | x | 190 | 0 to 14 | 0.90-1.20 | 60 | 0 to 14 | 0.90-1.20 |

### Supplementary Table 3. Effect of different levels of pollution on cardiac dyspnea for significant lag days for the 23 selected zones. Missing values are indicated with “x”. If some levels are missing, when possible, we defined novel intervals: good- and lightly+, corresponding to the maximal value reached by the corresponding pollutant in the specific zone as indicated in the table.

|  |  |  |  |  | NO_2_ | | | O_3_ | | | PM10 | | |
| --- | --- | --- | --- | --- | --- | --- | --- | --- | --- | --- | --- | --- | --- |
| Name of main city covered by correspondent zone | Cluster | Department | Number of CD  (6 years) | Pollution level | Value  (μg/m^3^) | Lag | RR  (95% CI) | Value  (μg/m^3^) | Lag | RR  (95% CI) | Value  (μg/m^3^) | Lag | RR  (95% CI) |
| Gap | 1 | 05 | 514 | Good | 90 | 0 to 14 | 0.80-1.30 | 80 | 0 to 14 | 0.50-1.50 | 25 | 0 to 14 | 0.80-1.20 |
|  |  |  |  | Moderate | 110  good- | 0 to 14 | 0.60-1.40 | 110 | 0 to 14 | 0.50-1.50 | 32 | 0 to 14 | 0.80-1.20 |
|  |  |  |  | Lightly | x | x | x | 150 | 0 to 14 | 0.50-2.00 | 45 | 0 to 14 | 0.60-1.40 |
|  |  |  |  | Heavily | x | x | x | 166 lightly+ | 0 to 14 | 0.50-3.00 | 60 | 0 to 14 | 0.40-1.80 |
| Draguignan | 1 | 83 | 468 | Good | 90 | 0 to 14 | 0.60-1.40 | 80 | 0 to 14 | 0.50-1.50 | 25 | 0 to 14 | 0.80-1.20 |
|  |  |  |  | Moderate | x | x | x | 110 | 0 to 14 | 0.50-1.50 | 32 | 0 to 14 | 0.80-1.20 |
|  |  |  |  | Lightly | x | x | x | 150 | 0 to 14 | 0.50-1.60 | 45 | 0 to 14 | 0.60-1.70 |
|  |  |  |  | Heavily | x | x | x | 184 | 0 to 14 | 0.50-2.30 | x | x | x |
| Pertuis | 1 | 84 | 342 | Good | 90 | 0 to 14 | 0.50-1.50 | 80 | 0 to 14 | 0.50-2.00 | 25 | 0 to 14 | 0.80-1.20 |
|  |  |  |  | Moderate | 107  good- | 0 to 14 | 0.40-2.00 | 110 | 0 to 14 | 0.50-2.00 | 32 | 0 to 14 | 0.80-1.20 |
|  |  |  |  | Lightly | x | x | x | 150 | 0 to 14 | 0.50-2.00 | 45 | 0 to 14 | 0.50-1.50 |
|  |  |  |  | Heavily | x | x | x | 190 | 0 to 14 | 0.50-2.50 | 55 | 0 to 14 | 0.50-3.00 |
| Manosque | 1 | 04 | 296 | Good | 90 | 0 to 14 | 0.60-1.40 | 80 | 0 to 14 | 0.90-1.80 | 25 | 0 to 14 | 0.70-1.30 |
|  |  |  |  | Moderate | x | x | x | 110 | 0 to 14 | 0.80-2.00 | 32 | 0 to 14 | 0.70-1.30 |
|  |  |  |  | Lightly | x | x | x | 150 | 0 to 14 | 0.80-2.00 | 45 | 0 to 14 | 0.50-1.50 |
|  |  |  |  | Heavily | x | x | x | 190 | 0 to 14 | 0.50-2.50 | 55 | 0 to 14 | 0.50-2.00 |
| St. Raphael | 2 | 83 | 498 | Good | 90 | 0 to 14 | 0.80-1.20 | 80 | 0 to 14 | 0.80-2.00 | 25 | 0 to 14 | 0.90-1.30 |
|  |  |  |  | Moderate | 128 | 0 to 14 | 0.60-1.80 | 110 | 0 to 14 | 0.60-2.00 | 32 | 0 to 14 | 0.90-1.30 |
|  |  |  |  | Lightly | x | x | x | 150 | 0 to 14 | 0.60-2.50 | 45 | 0 to 14 | 0.80-1.50 |
|  |  |  |  | Heavily | x | x | x | 187 | 0 to 14 | 0.50-3.00 | 55 | 0 to 14 | 0.50-2.00 |
| La Farlede | 2 | 83 | 277 | Good | 90 | 0 to 14 | 0.80-1.20 | 80 | 0 to 14 | 0.50-1.50 | 25 | 0 to 14 | 0.90-1.50 |
|  |  |  |  | Moderate | 126 | 0 to 14 | 0.60-1.80 | 110 | 0 to 14 | 0.50-1.50 | 32 | 0 to 14 | 0.90-1.50 |
|  |  |  |  | Lightly | x | x | x | 150 | 0 to 14 | 0.50-2.00 | 45 | 0 to 14 | 0.70-1.70 |
|  |  |  |  | Heavily | x | x | x | 190 | 0 to 14 | 0.50-3.50 | 55 | 0 to 14 | 0.50-2.00 |
| Digne Le Bains | 3 | 04 | 383 | Good | 82 | 0 to 14 | 0.40-1.60 | 80 | 0 to 14 | 0.60-1.50 | 25 | 0 to 14 | 0.80-1.20 |
|  |  |  |  | Moderate | x | x | x | 110 | 0 to 14 | 0.50-1.50 | 32 | 0 to 14 | 0.80-1.20 |
|  |  |  |  | Lightly | x | x | x | 150 | 0 to 14 | 0.50-2.00 | 45 | 0 to 14 | 0.70-2.50 |
|  |  |  |  | Heavily | x | x | x | 187 | 0 to 14 | 0.50-3.50 | 51 | 0 to 14 | 0.50-3.00 |
| Marseille | 4 | 13 | 5 111 | Good | 90 | 0 to 14 | 0.96-1.02 | 80 | 0 to 14 | 0.80-1.10 | 25 | 0 to 14 | 0.90-1.15 |
|  |  |  |  | Moderate | 130 | 0 to 14 | 0.96-1.04 | 110 | 0 to 14 | 0.80-1.10 | 32 | 0 to 14 | 0.90-1.15 |
|  |  |  |  | Lightly | 170 | 0 to 14 | 0.94-1.04 | 150 | 0 to 14 | 0.80-1.10 | 45 | 0 to 14 | 0.90-1.20 |
|  |  |  |  | Heavily | 210 | 0 to 14 | 0.94-1.04 | 190 | 0 to 14 | 0.80-1.30 | 60 | 0 to 14 | 0.80-1.30 |
| Aix en Provence | 4 | 13 | 708 | Good | 90 | 0 to 14 | 0.90-1.10 | 80 | 0 to 14 | 0.80-1.40 | 25 | 0 to 14 | 0.80-1.30 |
|  |  |  |  | Moderate | 130 | 0 to 14 | 0.80-1.10 | 110 | 0 to 14 | 0.70-1.40 | 32 | 0 to 14 | 0.80-1.30 |
|  |  |  |  | Lightly | 170 | 0 to 14 | 0.70-1.20 | 150 | 0 to 14 | 0.70-1.50 | 45 | 0 to 14 | 0.70-1.40 |
|  |  |  |  | Heavily | 210 | 0 to 14 | 0.60-1.30 | 190 | 0 to 14 | 0.70-1.80 | 60 | 0 to 14 | 0.40-2.00 |
| Nice | 4 | 06 | 4 144 | Good | 90 | 0 to 14 | 0.95-1.10 | 80 | 0 to 14 | 0.90-1.15 | 25 | 0 to 14 | 0.95-1.15 |
|  |  |  |  | Moderate | 130 | 0 to 14 | 0.90-1.12 | 110 | 0 to 14 | 0.90-1.15 | 32 | 0 to 14 | 0.95-1.15 |
|  |  |  |  | Lightly | 170 | 0 to 14 | 0.90-1.15 | 150 | 0 to 14 | 0.85-1.15 | 45 | 0 to 14 | 0.90-1.20 |
|  |  |  |  | Heavily | 210 | 0 to 14 | 0.85-1.15 | 190 | 0 to 14 | 0.70-1.20 | 55 | 0 to 14 | 0.80-1.30 |
| Cannes | 4 | 06 | 892 | Good | 90 | 0 to 14 | 0.95-1.05 | 80 | 0 to 14 | 0.80-1.20 | 25 | 0 to 14 | 0.80-1.10 |
|  |  |  |  | Moderate | 130 | 0 to 14 | 0.90-1.10 | 110 | 0 to 14 | 0.80-1.30 | 32 | 0 to 14 | 0.80-1.10 |
|  |  |  |  | Lightly | 170 | 0 to 14 | 0.80-1.25 | 150 | 0 to 14 | 0.70-1.40 | 45 | 0 to 14 | 0.70-1.20 |
|  |  |  |  | Heavily | x | x | x | 175  lightly+ | 0 to 14 | 0.60-1.60 | 57 | 0 to 14 | 0.60-1.60 |
| Toulon | 4 | 83 | 1 627 | Good | 90 | 0 to 14 | 0.95-1.03 | 80 | 0 to 14 | 0.60-1.30 | 25 | 0 to 14 | 0.80-1.20 |
|  |  |  |  | Moderate | 130 | 0 to 14 | 0.95-1.05 | 110 | 0 to 14 | 0.60-1.30 | 32 | 0 to 14 | 0.80-1.20 |
|  |  |  |  | Lightly | 170 | 0 to 14 | 0.90-1.15 | 150 | 0 to 14 | 0.60-1.40 | 45 | 0 to 14 | 0.70-1.30 |
|  |  |  |  | Heavily | 195  lightly+ | 0 to 14 | 0.90-1.20 | 190 | 0 to 14 | 0.60-1.80 | 55 | 0 to 14 | 0.60-1.50 |
| Grasse | 5 | 06 | 611 | Good | 90 | 0 to 14 | 0.90-1.20 | 80 | 0 to 14 | 0.70-1.50 | 25 | 0 to 14 | 0.80-1.20 |
|  |  |  |  | Moderate | 130 | 0 to 14 | 0.80-1.30 | 110 | 0 to 14 | 0.70-1.50 | 32 | 0 to 14 | 0.80-1.20 |
|  |  |  |  | Lightly | 147 | 0 to 14 | 0.70-1.40 | 150 | 0 to 14 | 0.60-1.70 | 45 | 0 to 14 | 0.60-1.30 |
|  |  |  |  | Heavily | x | x | x | 178  lightly+ | 0 to 14 | 0.50-2.50 | 55 | 0 to 14 | 0.40-1.60 |
| Seyne sur Mer | 5 | 83 | 862 | Good | 90 | 0 to 14 | 0.95-1.05 | 80 | 0 to 14 | 0.70-1.20 | 25 | 0 to 14 | 0.80-1.20 |
|  |  |  |  | Moderate | 130 | 0 to 14 | 0.90-1.10 | 110 | 0 to 14 | 0.70-1.40 | 32 | 0 to 14 | 0.80-1.20 |
|  |  |  |  | Lightly | 170 | 0 to 14 | 0.80-1.20 | 150 | 0 to 14 | 0.60-1.40 | 45 | 0 to 14 | 0.70-1.20 |
|  |  |  |  | Heavily | 196  lightly+ | 0 to 14 | 0.80-1.30 | 190 | 0 to 14 | 0.60-1.80 | 60 | 0 to 14 | 0.50-1.40 |
| Hyeres | 5 | 83 | 1001 | Good | 90 | 0 to 14 | 0.90-1.05 | 80 | 0 to 14 | 0.60-1.80 | 25 | 0 to 14 | 0.90-1.10 |
|  |  |  |  | Moderate | 130 | 0 to 14 | 0.75-1.10 | 110 | 0 to 14 | 0.60-1.80 | 32 | 0 to 14 | 0.90-1.10 |
|  |  |  |  | Lightly | 150 | 0 to 14 | 0.70-1.15 | 150 | 0 to 14 | 0.60-1.80 | 45 | 0 to 14 | 0.70-1.40 |
|  |  |  |  | Heavily | x | x | x | 190 | 0 to 14 | 0.60-1.90 | 55 | 0 to 14 | 0.60-1.80 |
| Frejus | 5 | 83 | 719 | Good | 90 | 0 to 14 | 0.90-1.10 | 80 | 0 to 14 | 0.60-1.30 | 25 | 0 to 14 | 0.80-1.20 |
|  |  |  |  | Moderate | 130 | 0 to 14 | 0.80-1.30 | 110 | 0 to 14 | 0.60-1.40 | 32 | 0 to 14 | 0.80-1.20 |
|  |  |  |  | Lightly | x | x | X | 150 | 0 to 14 | 0.50-1.40 | 45 | 0 to 14 | 0.60-1.20 |
|  |  |  |  | Heavily | x | x | x | 187 | 0 to 14 | 0.40-1.50 | 54 | 0 to 14 | 0.50-1.40 |
| Arles | 5 | 13 | 549 | Good | 90 | 0 to 14 | 0.90-1.10 | 80 | 0 to 14 | 0.50-1.50 | 25 | 0 to 14 | 0.80-1.10 |
|  |  |  |  | Moderate | 130 | 0 to 14 | 0.80-1.20 | 110 | 0 to 14 | 0.50-1.50 | 32 | 0 to 14 | 0.80-1.10 |
|  |  |  |  | Lightly | 159 | 0 to 14 | 0.70-1.40 | 150 | 0 to 14 | 0.50-1.50 | 45 | 0 to 14 | 0.70-1.20 |
|  |  |  |  | Heavily | x | x | x | 190 | 0 to 14 | 0.40-1.60 | 60 | 0 to 14 | 0.60-1.60 |
| Salon de Provence | 5 | 13 | 590 | Good | 90 | 0 to 14 | 0.90-1.10 | 80 | 0 to 14 | 0.80-1.60 | 25 | 0 to 14 | 0.80-1.20 |
|  |  |  |  | Moderate | 130 | 0 to 14 | 0.80-1.20 | 110 | 0 to 14 | 0.80-1.60 | 32 | 0 to 14 | 0.80-1.20 |
|  |  |  |  | Lightly | 155 | 0 to 14 | 0.70-1.40 | 150 | 0 to 14 | 0.80-1.70 | 45 | 0 to 14 | 0.80-1.50 |
|  |  |  |  | Heavily | x | x | x | 190 | 0 to 14 | 0.60-2.00 | 60 | 0 to 14 | 0.50-2.50 |
| La Ciotat | 5 | 13 | 623 | Good | 90 | 0 to 14 | 0.90-1.10 | 80 | 0 to 14 | 0.70-1.60 | 25 | 0 to 14 | 0.80-1.20 |
|  |  |  |  | Moderate | 130 | 0 to 14 | 0.80-1.40 | 110 | 0 to 14 | 0.70-1.60 | 32 | 0 to 14 | 0.80-1.20 |
|  |  |  |  | Lightly | 150 | 0 to 14 | 0.70-1.60 | 150 | 0 to 14 | 0.60-1.60 | 45 | 0 to 14 | 0.80-2.00 |
|  |  |  |  | Heavily | x | x | x | 190 | 0 to 14 | 0.40-1.80 | 60 | 0 to 14 | 0.50-4.00 |
| Le Luc | 5 | 83 | 301 | Good | 90 | 0 to 14 | 0.90-1.30 | 80 | 0 to 14 | 0.50-1.50 | 25 | 0 to 14 | 0.80-1.20 |
|  |  |  |  | Moderate | 130 | 0 to 14 | 0.50-2.00 | 110 | 0 to 14 | 0.40-1.50 | 32 | 0 to 14 | 0.80-1.20 |
|  |  |  |  | Lightly | 148 | 0 to 14 | 0.50-2.50 | 150 | 0 to 14 | 0.30-1.50 | 45 | 0 to 14 | 0.50-1.50 |
|  |  |  |  | Heavily | x | x | x | 188 | 0 to 14 | 0.10-2.00 | 54 | 0 to 14 | 0.50-2.50 |
| Cavaillon | 6 | 84 | 369 | Good | 90 | 0 to 14 | 0.80-1.20 | 80 | 0 to 14 | 0.50-1.50 | 25 | 0 to 14 | 0.80-1.20 |
|  |  |  |  | Moderate | 125 | 0 to 14 | 0.60-1.80 | 110 | 0 to 14 | 0.50-1.50 | 32 | 0 to 14 | 0.60-1.20 |
|  |  |  |  | Lightly | x | x | x | 150 | 0 to 14 | 0.50-2.00 | 45 | 0 to 14 | 0.60-1.20 |
|  |  |  |  | Heavily | x | x | x | 190 | 0 to 14 | 0.50-2.50 | 60 | 0 to 14 | 0.40-1.60 |
| Avignon | 6 | 84 | 990 | Good | 90 | 0 to 14 | 0.90-1.10 | 80 | 0 to 14 | 0.80-1.20 | 25 | 0 to 14 | 0.80-1.20 |
|  |  |  |  | Moderate | 130 | 0 to 14 | 0.80-1.20 | 110 | 0 to 14 | 0.80-1.30 | 32 | 0 to 14 | 0.80-1.20 |
|  |  |  |  | Lightly | 148 | 0 to 14 | 0.70-1.20 | 150 | 0 to 14 | 0.80-1.50 | 45 | 0 to 14 | 0.80-1.20 |
|  |  |  |  | Heavily | x | x | x | 190 | 0 to 14 | 0.80-2.00 | 60 | 0 to 14 | 0.80-1.80 |
| Orange | 6 | 84 | 509 | Good | 90 | 0 to 14 | 0.90-1.10 | 80 | 0 to 14 | 0.80-1.40 | 25 | 0 to 14 | 0.80-1.20 |
|  |  |  |  | Moderate | 130 | 0 to 14 | 0.70-1.30 | 110 | 0 to 14 | 0.80-1.40 | 32 | 0 to 14 | 0.80-1.20 |
|  |  |  |  | Lightly | x | x | x | 150 | 0 to 14 | 0.60-1.60 | 45 | 0 to 14 | 0.80-1.20 |
|  |  |  |  | Heavily | x | x | x | 190 | 0 to 14 | 0.40-1.80 | 60 | 0 to 14 | 0.60-1.60 |

Supplementary Figure 1. Time series trends for each pollutant and meteorological variables in Région Sud in 2013 to 2018 are represented.

Supplementary Figure 2. The average values of single pollutant by zone are reported.

Supplementary Figure 3. The correlations between air pollution and meteorological factors are presented.

Supplementary Figure 4. Exposure curves for pollutants showing the trend of RR on CD by lag on the entire region , 2013–2018.

**Supplementary Figure 5.** DLNM by singular zones, only significant results for the selected 23 zones are reported. Lag-specific effect on CD events for increases of NO2 (A), O3 (B) and PM10 (C) over the thresholds indicated in the legend in the plot.
